# Relative deprivation and depressive symptoms among Chinese migrant children: The impacts of self-esteem and belief in a just world

**DOI:** 10.3389/fpubh.2022.1008370

**Published:** 2022-10-20

**Authors:** Meng Xiong, Zhiqin Hu

**Affiliations:** ^1^School of Education and Sports Sciences, Yangtze University, Jingzhou, China; ^2^Department of Psychology, University of Edinburgh, Edinburgh, United Kingdom

**Keywords:** migrant children, relative deprivation, self-esteem, belief in a just world, depressive symptoms

## Abstract

**Background:**

Studies have shown that relative deprivation is a risk factor for depressive symptoms, but the underlying mechanisms are not yet clarified. Thus, this study formulated a moderated mediation model to investigate the mediating role of self-esteem and the moderating role of belief in a just world between relative deprivation and depressive symptoms among rural-to-urban migrant children.

**Methods:**

A sample of 1,076 Chinese migrant children (*M*_age_ = 12.25 years, *SD* = 1.66) completed measurements of relative deprivation, self-esteem, belief in a just world, and depressive symptoms. Furthermore, the mediating mechanism and moderating effect of the study were explored with the SPSS PROCESS macro (Models 4 and 7).

**Results:**

The results showed a significant positive association between relative deprivation and depressive symptoms, with self-esteem partially mediating this association. Moreover, belief in a just world moderated the association between relative deprivation and self-esteem. Namely, the indirect effect of self-esteem was moderated by belief in a just world. Specifically, the mediating effect was stronger for migrant children with higher levels of belief in a just world.

**Conclusion:**

These findings broaden our knowledge of how and when relative deprivation influences depressive symptoms among migrant children. Therefore, appropriate measures should be taken to prevent and manage migrant children' depression and provide them with corresponding guidance. Some measures could be taken by schools and educators to help migrant children with high relative deprivation in improving their self-esteem and belief in a just world, such as self-reference tasks and psychological intervention programs.

## Introduction

During the past several decades, China has experienced an unprecedented migration from rural to urban regions due to its rapidly developing economy and urbanization (1). A side effect of the large scale of migration is that children have moved with their parents en masse from rural areas to urban regions (2). Rural-to-urban migrant children are described as children, under 18 years old, who have left their original residence and migrated to destination cities for more than 6 months (3). Based on the data from China's Ministry of Education in 2019, there were approximately 14 million migrant children in elementary and junior high schools (4). Researchers have documented that rural-to-urban migration decreases children's well-being while increasing problem behaviors (3, 5). In particular, rural-to-urban migrant children are more susceptible to depression compared to their non-migrant peers (6). Evidence has shown that depression is prevalent among adolescents in the twenty first century (7, 8). Furthermore, a study revealed that about 21% of the 677 Chinese migrant children were diagnosed with depression (9). Depression can lead to various undesirable consequences in psychological and social adjustment (10, 11). Therefore, scrutinizing the risk factors and relevant mechanisms for rural-to-urban migrant children's depression is necessary for prevention and intervention efforts.

Relative deprivation refers to a subjective cognition and affective experience with negative emotions, such as anger and resentment, when people perceive that they are in a disadvantaged position through horizontal or vertical comparison (12, 13). It might be a risk factor for depressive symptoms among migrant children. Studies show that relative deprivation is significantly positively correlated with depression among Chinese college students (14, 15). However, as previous studies have focused on the general population, little is known about the association between relative deprivation and depressive symptoms in migrant children. Additionally, prior research has concentrated on the direct relationship between relative deprivation and depressive symptoms, with the underlying mediating (i.e., how relative deprivation is associated with depressive symptoms) and moderating mechanisms (i.e., when relative deprivation is associated with depressive symptoms) remaining unclear. Thus, to address these gaps, this study proposed a moderated mediation model to reveal the underlying mechanisms of this relationship in rural-to-urban migrant children. Given that high self-esteem is a mental mechanism of individuals in their adaptation to the sociocultural environment (16), this study tested self-esteem as a mediator to clarify the influencing mechanisms of relative deprivation on depressive symptoms. Moreover, prior studies have indicated that belief in a just world is a powerful buffer that mitigates the adverse effects of risk factors on left-behind children's mental health and problem behaviors (17). Thus, belief in a just world, which is defined as the believe that people live in a world where they generally receive rewards and/or punishments they deserve (18), might act as a moderator in this study.

### Relative deprivation and depressive symptoms

Research has also indicated that relative deprivation leads to various undesirable outcomes, such as depression (19), anxiety disorders (20), and aggressive behavior (13). Compared with their non-migrant counterparts, children who migrate with their parents from rural to urban areas are more likely to encounter relative deprivation due to the household registration system (or *hukou*) in China (6, 21). Specifically, many cities deny rural-to-urban migrant children access to public schools because public schools' admittance standards are contingent upon local legal residency. Although migrant families relocate to urban areas, they still maintain their rural residential status (6).

Theoretical and empirical research has revealed the relationship between relative deprivation and depressive symptoms. First, considering that relative deprivation contains two components—subjective cognition and affective experience (13)—it may affect depression through both cognition and emotion. Individuals with high relative deprivation experience negative emotions, including indignation, sadness, and disgruntlement (22); these emotions are significant indicators of depression. Thus, relative deprivation may influence depressive symptoms. Additionally, the cognitive model of depression (23) highlighted the role of cognitive vulnerabilities in the onset and development of depression. Namely, unreasonable cognition caused by relative deprivation may aggravate depression in individuals. Second, the relative deprivation theory (24) posits that disadvantaged groups, such as migrant children, experience relative deprivation in upward social comparison. This feeling of deprivation not only results in interpersonal problems, but also damages individual psychological health, which is a risk factor for depressive symptoms (25). More importantly, depression is a crucial dimension of psychological well-being (26). Accordingly, relative deprivation enhances the likelihood of depressive symptoms. Third, cross-sectional and longitudinal studies have also demonstrated that higher levels of relative deprivation are associated with higher levels of depression. For example, individuals with relative deprivation have more depressive symptoms (27). Another longitudinal study by Schmitt et al. (28) documented that earlier relative deprivation could predict later poor mental health (e.g., depression) in a sample of 1276 East Germans over the course of 4 years. Additionally, a meta-analysis showed that relative deprivation predicted the level of depression, with *r* = 0.173 (29). Thus, based on relevant theories and empirical studies, this study assumed that relative deprivation is positively associated with depressive symptoms among migrant children (Hypothesis 1).

### Self-esteem as a mediator

Self-esteem is described as one's sense of self-worth, reflecting a positive or negative assessment and attitude toward oneself (30, 31). Multiple studies have revealed that various factors could affect self-esteem, such as family socioeconomic status (32), cyberbullying (33), and social comparison (34). Relative deprivation is also an important inducer of self-esteem as its core psychological process is social comparison (35). Social comparison theory (36, 37) states that upward social comparison is a critical factor leading to low self-esteem. Moreover, when individuals feel this kind of unfavorable socioeconomic status, they may have more negative self-evaluation and tend to conduct self-aggression (38), which decreases the level of self-esteem (39). Compelling evidence suggests that relative deprivation is inversely correlated with self-esteem. Studies on the direct association between relative deprivation and self-esteem have also verified that relative deprivation is a risk factor for low self-esteem (29). For instance, an experimental study by Walker (40) found that personal relative deprivation lowered self-esteem. Further, both cross-sectional and longitudinal research found that relative deprivation was negatively linked to self-esteem (41, 42).

Depression is one of the major adverse consequences of low self-esteem. There are two reasons supporting this inference. Primarily, self-esteem is an important predictor of mental health (43); simultaneously, depression is also a crucial dimension of psychological well-being (44). Besides, according to the vulnerability and scar models of low self-esteem and depression (45, 46), low self-esteem is a vulnerable diathesis of depression. In other words, high level of self-esteem could protect individuals from the influence of depressive symptoms, whereas low self-esteem increases the risk of depressive symptoms. Second, a large body of empirical research suggests that reducing the level of self-esteem predicts more depressive symptoms. Notably, a longitudinal study by Fan et al. (47) suggested that low self-esteem at baseline was a risk factor for depression at a 9 month follow-up in Chinese migrant children. Additionally, a series of cross-sectional studies found that depressive symptoms deteriorated with the aggravation of low self-esteem (48, 49). Thus, we can conclude that low self-esteem affects depressive symptoms. As mentioned above, relative deprivation negatively predicts self-esteem, which leads to more depressive symptoms. Consistent with this theoretical framework, an empirical study showed that self-esteem partially mediated the association between relative deprivation and aggression among young male migrant workers (39). Overall, it is reasonable to assume that self-esteem serves as a mediator. Therefore, this study hypothesized that self-esteem mediates the relationship between relative deprivation and depressive symptoms (Hypothesis 2).

### Belief in a just world as a moderator

Although relative deprivation may affect self-esteem and depressive symptoms, it is impossible for all migrant children to be equally influenced. Thus, it is important to verify moderators that may strengthen or weaken the relationship between relative deprivation and unfavorable consequences. Though most people possess belief in a just world, the degree of belief may vary between individuals (50). Belief in a just world works as an effective coping mechanism that buffers the harmful effects of stress against depression (51). High belief in a just world has beneficial outcomes for individuals, such as improving mood and maintaining subjective well-being (52). Furthermore, it can facilitate self-esteem (53); a study revealed that belief in a just world is positively associated with self-esteem (54). At the same time, the just-world theory (55, 56) indicates that individuals with higher belief in a just world are more likely to prefer fairness. Accordingly, belief in a just world may attenuate the adverse effects of negative factors on psychological well-being. For instance, a study by Kim and Park (57) showed that belief in a just world moderated the relationship between perceived sex discrimination and self-esteem, with the relationship being more potent for married Korean working women with a higher belief in a just world. Moreover, high belief in a just world weakened the impact of relative deprivation on life satisfaction (58). Thus, belief in a just world may play a buffering role and alleviate the detrimental consequences of relative deprivation (e.g., low self-esteem).

The risk and protective factor models (59) identified that the undesirable effect of one risk factor (e.g., relative deprivation) on its outcomes (e.g., self-esteem) may be reduced by a protective factor (e.g., belief in a just world). Notably, migrant children with high belief in a just world are more likely to have a sense of security and control (60), which could decrease the impact of relative deprivation and improve the level of self-esteem. Comparatively, low belief in a just world refers to a more negative perception of the world (18). At this point, the protective effect of a low belief, compared with a high belief in a just world, is not significant. Therefore, it is hypothesized that belief in a just world could protect self-esteem from the detrimental effects of relative deprivation (Hypothesis 3).

**Figure 1:**
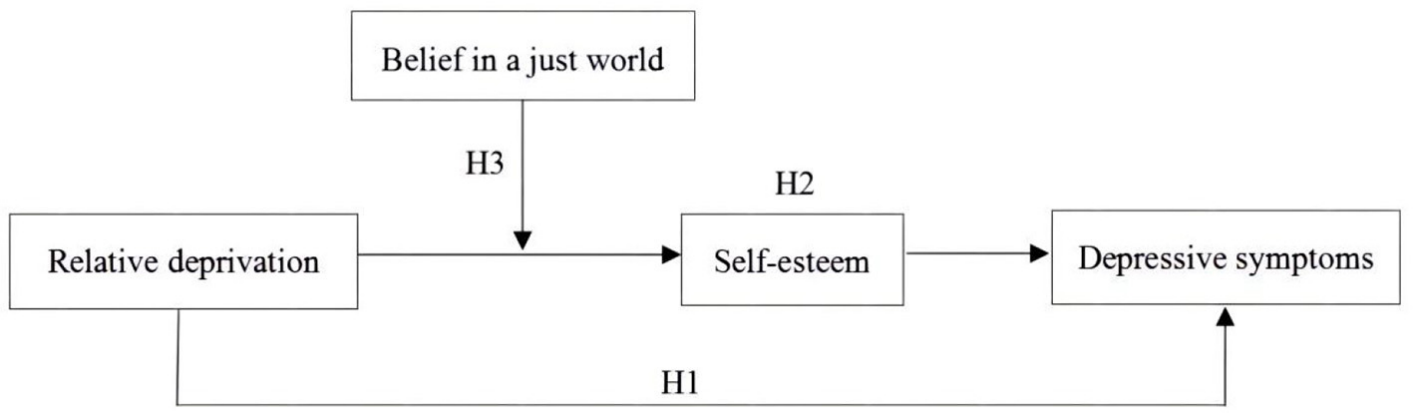
Moderated mediation model of the present study. Of these, H1 refers to that relative deprivation is positively associated with depressive symptoms. H2 represents that self-esteem mediate the association between relative deprivation and depressive symptoms. H3 refers to that belief in a just world moderate the relation between relative deprivation and self-esteem.

In summary, based on the relative deprivation theory and the risk-protective factor models, this study constructed a moderated mediation model to investigate whether relative deprivation is indirectly related to depressive symptoms through self-esteem and whether the indirect relationship is moderated by belief in a just world. The aims of this study were 3-fold: (a) to examine whether relative deprivation is positively correlated with depressive symptoms in Chinese migrant children; (b) to test whether self-esteem plays an intermediary role in the relationship between relative deprivation and depressive symptoms; and (c) to explore whether belief in a just world plays a regulatory role in the relationship between relative deprivation and self-esteem. Figure 1 illustrates the moderated mediation model.

## Materials and methods

### Participants

The migrant children who participated in the study were recruited via a cluster sampling from three primary schools (4th−6th grade) and three junior high schools (7th−9th grade) in Fujian Province, China. The eligibility criteria for migrant children included: (a) school-aged children born in rural regions without an urban *hukou*; (b) those ever living in their rural hometowns; (c) those who had accompanied their parents to the destination cities; and (d) those who had been living in the destination cities for more than 6 months (61). Migrant children completed relevant measurements in a classroom after acquiring permission from school principals and written informed consent from their parents. In this process, trained interviewers ensured standard instructions and provided necessary clarifications for migrant children. Participants completed paper-and-pencil questionnaires, which took ~20 min. All participants received ballpoint pens as rewards after completing the survey. The procedures were approved by the Ethics Committee of Academic Research at the corresponding author's institution.

A total of 1,167 migrant children's responses were collected. The responses were deleted if more than three items were left unanswered or were answered in the same way. Meanwhile, the mean method was used for the remaining questionnaires to handle missing values. Finally, 1,076 surveys were validated, which accounted for 92.2% of the total administered. The average age was 12.25 years (*SD* = 1.66), with an age range of 10–15 years. Of the 1,076 participants, 547 (50.8%) were male participants, 524 (48.7%) were female participants, and five students did not report their sex. There were 626 (58.34%) elementary school students and 447 (41.66%) junior high school students. Two hundred and twenty-eight (21.33%) participants were the only children in their families and 841 (78.67%) participants had siblings. Regarding the parent educational background of migrant children, 229 (21.44%) fathers had a primary school level of education or below, 418 (39.14%) had a junior high school level of education, 180 (16.85%) reported a senior high school level of education, 79 (7.4%) had a university degree or above, and 162 (15.17%) reported their education level as unknown. Additionally, 374 (34.99%) mothers had a primary school level of education or below, 365 (34.14%) had a junior high school level of education, 100 (9.35%) had a senior high school level of education, 62 (5.8%) had a university degree or above, and 168 (15.72%) reported their education level as unknown. In terms of monthly family income, 165 (15.79%), 463 (44.3%), and 417 (39.9%) had an average monthly family income of <2,000 yuan, between 2,000 and 5,000 yuan, and more than 5,000 yuan, respectively (31 migrant children did not report).

### Measures

#### Relative deprivation

This was measured using the Relative Deprivation Scale for Migrant Children (62), which had good reliability and validity for Chinese migrant children (63). The scale included 20 items categorized into four dimensions: cognition of individual relative deprivation, the emotion of individual relative deprivation, cognition of group relative deprivation, and the emotion of group relative deprivation. The example items include “*Are you satisfied with this situation*?” and “*How satisfied are you with this situation*?” This scale referred to five aspects of a real situation: family economic status, housing conditions and living environment, stability of residence, opportunities for developing strong points, and the degree of parental involvement in tutoring homework. The items of the cognitive dimension ranged from 1 (*very good*) to 7 (*very bad*), and the items of the emotional dimension ranged from 1 (*very satisfied*) to 7 (*extremely unsatisfied*). Higher scores indicate higher level of relative deprivation. In this study, Cronbach's α for the scale was 0.92.

#### Self-esteem

This was assessed using the Rosenberg Self-Esteem Scale (30). In the Chinese revised version, the 8th question (e.g., “*I wish I could earn more respect for myself* ”) was excluded because of cultural differences (38). Participants rated nine items (e.g., “*On the whole, I am satisfied with myself* ;” “*I feel that I have a number of good qualities*”) on a 4-point Likert scale (1= *strongly disagree*, 4= *strongly agree*) with higher scores indicating higher self-esteem. The reliability and validity of the scale have been validated among Chinese migrant children (64). In this study, Cronbach's α for the scale was 0.80.

#### Belief in a just world

The Scale of Belief in a Just World (65) has been widely used to measure belief in a just world. This scale has been used in Chinese migrant children, reporting good reliability and validity (66). The 13 self-report items were divided into two dimensions: personal belief in a just world (e.g., “*I feel that my efforts are noticed and rewarded*”) and general belief in a just world (e.g., “*I feel that I get what I deserve*”). Participants responded to items on a 6-point Likert scale, with values ranging from 1 (*strongly disagree*) to 6 (*strongly agree*). Higher scores indicate a greater belief in a just world. In this study, Cronbach's α for the scale was 0.88.

#### Depressive symptoms

We used the Center for Epidemiologic Studies-Depression Scale (67). The Chinese version has been widely used in Chinese research (68) and has good reliability and validity (69). It comprised 20 items (e.g., “*I did not feel like eating*; *my appetite was poor*;” “*I thought my life had been a failure*”), measuring six symptoms of depression (e.g., depressive mood, guilt/unworthiness, and loss of appetite). Participants responded on a 4-point Likert scale ranging from 1 (*rarely or none of the time* [*less than one day*]) to 4 (*most or all of the time*), with higher scores indicating more symptoms of depression. In this study, Cronbach's α for the scale was 0.87.

### Statistical analysis

All statistical analyses were conducted using Statistical Package for the Social Sciences (SPSS) 25.0. First, descriptive statistics and correlation analyses were conducted to explore the potential associations between relative deprivation, self-esteem, belief in a just world, and depressive symptoms. Second, the SPSS PROCESS macro (Models 4 and 7) suggested by Hayes (70) was performed to further test the mediating role of self-esteem and the moderating role of belief in a just world. It has been extensively used in previous research to examine complex models, including moderated mediation and mediated moderation models (57). Moreover, considering that there are significant age and/or sex differences in individuals' relative deprivation (14) and depression (71), we included age and sex as covariates in all analyses.

## Results

### Comparison of depressive symptoms levels in different demographic characteristics

There are significant differences in the scores of depressive symptoms among migrant children in terms of grade level, father's educational background, and monthly family income, but there are no significant differences in terms of sex, only child status, and mother's educational background in Table 1. In terms of grade, the depressive scores of junior high school students were significantly higher than those of elementary school students (*p* < 0.05). In terms of father's educational background, the migrant children whose fathers' education level was primary school and below had the highest depressive scores, and the posttest (LSD) suggests that the depressive scores are significantly higher than those of fathers' with other educational levels (*p* < 0.05). In terms of monthly family income, the depressive symptoms of migrant children with < 2,000 yuan of monthly family income were significantly higher than those of other levels (*p* < 0.001).

**Table 1:** Comparison of depressive symptoms scores of the migrant children with different demographic characteristics.

**Variable**	**Project**	**Number of people**	**Depressive symptoms**	***t*/*F***	***p***
Sex	Male	547	37.34 ± 10.82	1.51	0.13
	Female	524	36.36 ± 10.53		
Grade	Elementary school students	626	36.89 ± 10.33	−2.10	0.04
	Junior high school students	447	38.27 ± 10.97		
Only child status	Only child	228	37.64 ± 11.73	0.20	0.84
	Non-only-child	841	37.47 ± 10.31		
Father education	Primary school education or below	229	39.29 ± 10.76	2.90	0.02
	Junior high school education	418	37.48 ± 10.41		
	Senior high school education	180	36.40 ± 10.84		
	University degree or above	79	37.30 ± 10.24		
	Unclear	162	36.06 ± 10.48		
Mother education	Primary school education or below	374	38.48 ± 10.32	1.58	0.18
	Junior high school education	365	36.57 ± 10.56		
	Senior high school education	100	37.58 ± 11.46		
	University degree or above	62	36.87 ± 11.15		
	Unclear	168	37.32 ± 10.40		
Monthly family income	< 2,000 yuan	165	40.35 ± 11.18	7.74	< 0.001
	2,000–5,000 yuan	463	36.74 ± 10.25		
	>5,000 yuan	417	36.74 ± 10.53		

### Preliminary results

The results of the means, standard deviations, and Pearson correlations between migrant children's relative deprivation, self-esteem, belief in a just world, and depressive symptoms are presented in Table 2. Relative deprivation was negatively associated with self-esteem and belief in a just world, and positively associated with depressive symptoms. Self-esteem was positively linked to belief in a just world and negatively linked to depressive symptoms. Belief in a just world was negatively correlated with depressive symptoms.

**Table 2:** Descriptive statistics and correlations among key variables.

**Variables**	***M***	***SD***	**1**	**2**	**3**	**4**	**5**	**6**
1.Age	12.25	1.66	-					
2.Sex	-	-	−0.01	-				
3.Relative deprivation	3.22	0.96	0.24**	−0.01	-			
4.Self-esteem	2.88	0.51	−0.08**	0.05	−0.27**	-		
5.Belief in a just world	4.17	0.94	−0.17**	0.04	−0.37**	0.34**	-	
6.Depressive symptoms	1.84	0.53	0.07*	−0.05	0.21**	−0.55**	−0.32**	-

N = 1,076. Sex was a virtual variable: 0 represented female students, 1 represented male students.

^*^p < 0.05,

^**^p < 0.01.

### Mediating effect of self-esteem

As shown in Table 3, when controlling for the age and sex of migrant children, relative deprivation was positively correlated with depressive symptoms (*B* = 0.21, *p* < 0.001), and negatively correlated with self-esteem (*B* = −0.27, *p* < 0.001). Self-esteem was negatively correlated with depressive symptoms (*B* = −0.54, *p* < 0.001). Additionally, relative deprivation was still positively correlated with depressive symptoms (*B* = 0.06, *p* < 0.01) when relative deprivation and self-esteem predicted depressive symptoms together. The bias-corrected percentile bootstrap method revealed that the mediating effect of relative deprivation on depressive symptoms through self-esteem was 0.15, SE was 0.02, and its 95% confidence interval was [0.11, 0.18] (did not contain 0). The direct (0.06) and indirect (0.15) effect accounted for ~28.57 and 71.42% of the total effect, respectively. Namely, self-esteem partially mediated the relationship between relative deprivation and depressive symptoms among migrant children. Therefore, Hypothesis 2 was supported.

**Table 3:** Mediating effect of self-esteem between relative deprivation and depressive symptoms.

**Predictors (IV)**	**Model 1**		**Model 2**		**Model 3**	
	**(criterion: depressive symptoms)**		**(criterion: self-esteem)**		**(criterion: depressive symptoms)**	
	***B***	***t***	***B***	***t***	***B***	***t***
Age	0.01	0.67	−0.01	−0.55	0.01	0.45
Sex	0.09	1.61	−0.11	−1.82	0.04	0.76
Relative deprivation	0.21***	6.58***	−0.27	−8.77***	0.06	2.20**
Self-esteem					−0.54	−20.09***
*R* ^2^	0.05		0.08		0.31	
*F*	17.16***		29.39***		118.76***	

N = 1,076.

^**^p < 0.01,

^***^p < 0.001.

### Moderating effect of belief in a just world

As noted, Hypothesis 3 predicted that belief in a just world would moderate the first half of the mediating relationship between relative deprivation and depressive symptoms through self-esteem. As shown in Table 4, after controlling for age and sex, relative deprivation negatively predicted self-esteem (*B* = −0.17, *p* < 0.001), and the interaction of relative deprivation and belief in a just world showed significant effects on self-esteem (*B* = −0.05, *p* < 0.05). This finding suggests that the relationship between relative deprivation and self-esteem was moderated by the belief in a just world, that is, the mediating role of self-esteem was moderated by belief in a just world.

**Table 4:** Moderated mediation analysis results with belief in a just world as a moderator.

**Predictors (IV)**	**Depressive symptoms**			**Self-esteem**		
	***B***	**SE**	***t***	***B***	**SE**	***t***
Age	0.007	0.02	0.45	0.002	0.02	0.09
Sex	0.04	0.05	0.76	−0.09	0.06	−1.6
Relative deprivation	0.06	0.03	2.21**	−0.17	0.03	−5.43***
Self-esteem	−0.54	0.03	−20.09***	-	-	-
Belief in a just world	-	-	-	0.29	0.03	9.31***
Relative deprivation × Belief in a just world	-	-	-	−0.05	0.03	−2.05*
*R* ^2^	0.31			0.15		
*F*	118.76***			36.92***		

N = 1,076.

^*^p < 0.05,

^**^p < 0.01,

^***^p < 0.001.

**Figure 2:**
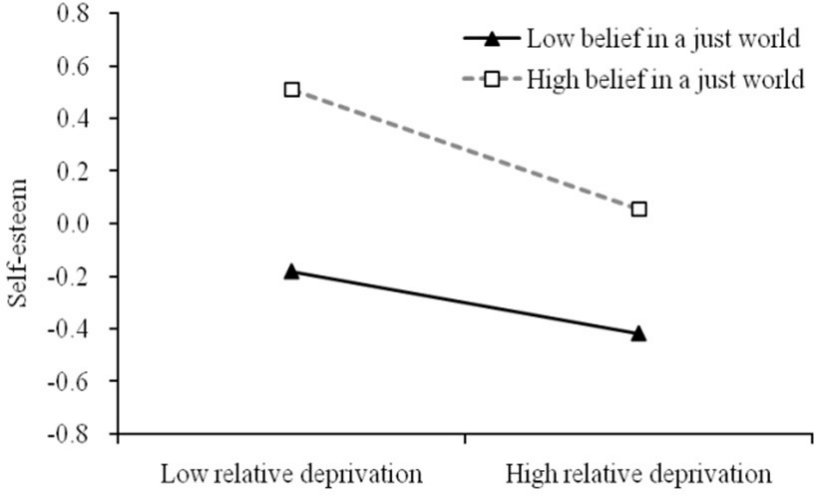
Moderator role of belief in a just world between relative deprivation and self-esteem.

**Figure 3:**
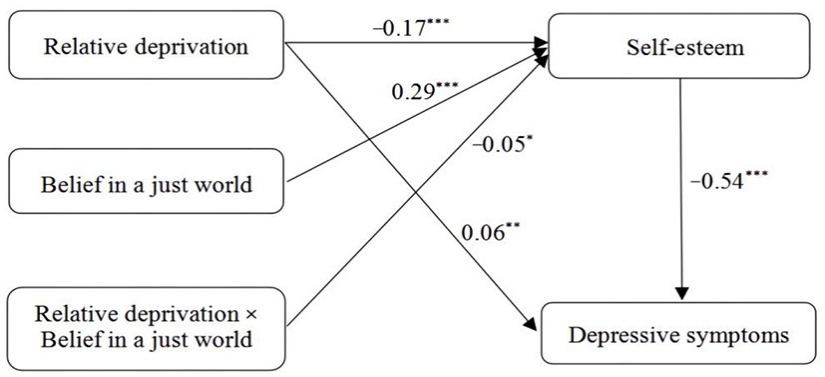
The moderated mediation model with key results. ^*^p < 0.05, ^**^p < 0.01, ^***^p < 0.001.

Moreover, to understand the moderating effect better, we conducted a simple slopes analysis (72). The simple slopes test reflected the association between relative deprivation and self-esteem at two levels of belief in a just world (1 SD below the mean and 1 SD above the mean). Figure 2 shows that relative deprivation was a stronger negative predictor of self-esteem for migrant children who had a higher level of belief in a just world (*simple slope* = −0.22, *t* = −5.42, *p* < 0.001) than for migrant children who had lower beliefs in a just world (*simple slope* = −0.12, *t* = −2.87, *p* < 0.01). The bias-corrected percentile bootstrap method further showed the indirect effect of relative deprivation on depressive symptoms via self-esteem was moderated by belief in a just world. Specifically, the mediating effect of relative deprivation on depressive symptoms via self-esteem was stronger at high belief in a just world (effect size = 0.12, 95% CI = [0.07, 0.17]), but weaker in low belief in a just world (effect size = 0.06, 95% CI = [0.02, 0.11]). Thus, Hypothesis 3 was supported. Figure 3 distinctly describes the moderated mediation model and key path coefficients for migrant children.

## Discussion

The moderated mediation model emphasized relative deprivation as a vital predictor of depressive symptoms, which illustrates rural-to-urban migrant children with a high level of relative deprivation may be at a high risk of depressive symptoms. This result has gained extensive attention and empirical support (27, 73). Besides, the current study showed that self-esteem played a mediating role between relative deprivation and depressive symptoms. Meanwhile, the first half of the mediation process was moderated by belief in a just world. Specifically, with the effect being stronger for migrant children with high belief in a just world than for those with low belief in a just world. These findings deepen our understanding of the underlying relationship mechanisms between relative deprivation and depressive symptoms.

### Association between relative deprivation and depressive symptoms

Previous studies have shown that the level of relative deprivation at baseline predicted depression at a 1.5 year follow-up period in single-parent children (41). Also, recent research has indicated that relative deprivation is positively related to depression (74). Consistent with previous studies (15, 27, 75), this study found that relative deprivation significantly predicted depression; in other words, relative deprivation was a risk factor for depressive symptoms among migrant children. Two possible explanations have been postulated to explain the relationship between relative deprivation and depressive symptoms, namely cognition and emotion. First, relative deprivation plays a significant cognitive vulnerability role for health (22) and is likely to create a strong sense of hopelessness, which might lead to depression (76). Thus, this result coincides with the cognitive model of depression. Second, migrant children exposed to a high level of relative deprivation generate undesirable emotional experiences (13, 77), such as anger, anxiety, and dejection. This accumulation of negative emotions is one of the major reasons for depression (78).

The social rank theory of depression (79) states that subordination or inferiority due to relative deprivation induces depression. Compared with advantageous groups (non-migrant children), rural-to-urban migrant children are more likely to develop deep hopelessness and fatalism about their educational attainment and future achievements when they perceive that they are relatively deprived (73). These feelings and negative emotion lower migrant children's expectations, thereby resulting in more depressive symptoms. Therefore, migrant children suffering from relative deprivation might experience more depressive symptoms.

### Mediating effect of self-esteem

Our study found that self-esteem partially mediated the link between relative deprivation and depressive symptoms among migrant children. This result is congruent with previous empirical studies and our assumption that self-esteem has a mediating role in this relationship (31, 32). Self-esteem was a significant mechanism for explaining why and how relative deprivation influenced depressive symptoms. First, regarding the first stage of the mediation effect, higher relative deprivation was correlated with lower self-esteem. Migrant children feel inequality and deprivation in family socioeconomic status, welfare guarantee, and educational opportunity during upward comparison to their urban peers, which induces relative deprivation, and thereby underestimation of ego values, triggering negative self-assessment and cognition. Sociometer theory (80) highlights the social nature of self-esteem and presumes that it serves as a sociometer—an internal scale of others' evaluations of oneself. Consequently, relative deprivation had a significant and negative effect on self-esteem in rural-to-urban migrant children. Moreover, an experimental study conducted by Kim et al. (81) confirmed that personal relative deprivation negatively predicted self-esteem.

Second, low self-esteem was correlated with more depressive symptoms in the second stage of the mediation model. This finding is consistent with a recent study by Bang et al. (31), which revealed that high self-esteem reduced the occurrence rate of depression. One possible reason is that high self-esteem can protect individuals against the harmful impact of depression (69). Under these circumstances, migrant children with high self-esteem have a distinct self-concept and favorable assessment (82); thus, the impact of negative factors is likely to be attenuated. Additionally, high self-esteem is linked to good mental and behavioral health, such as high self-efficacy (83) and prosocial behavior (84). Conversely, migrant children with low self-esteem have more unfavorable self-cognitions and self-evaluations as well as self-aggression, which are vulnerable factors of depression (85). We think that rural-to-urban migrant children will feel their own inferior family socioeconomic status during urban integration, which will induce a sense of frustration as well as deprivation, and may further weaken their self-assessment or the level of self-esteem, thereby increasing depressive symptoms. Therefore, our hypothesis that self-esteem plays a mediating role between relative deprivation and depressive symptoms among migrant children was supported.

### Moderating effect of belief in a just world

This study demonstrated that belief in a just world moderated the first half of the mediating relationship between relative deprivation and depressive symptoms through self-esteem. In line with the risk-buffering hypothesis (86), the belief in a just world might alleviate the adverse impact of relative deprivation on self-esteem. This result aligns with our assumption that belief in a just world generates a moderating effect; that is, the mediating effect of self-esteem was moderated by belief in a just world. Specifically, the relationship between relative deprivation and self-esteem was stronger for rural-to-urban migrant children with high belief in a just world than for those with low belief in a just world.

Based on prior studies (50, 57), there are several reasons why belief in a just world plays a moderating role between relative deprivation and self-esteem. First, the justice motivation theory (55) asserts that belief in a just world provides numerous psychological resources to prevent disadvantaged groups (e.g., migrant children) from the negative effects of stressful and unfair events. In other words, the more resources migrant children have, the more capacity they have for facing unfairness (57). Second, individuals with high belief in a just world could cope with harmful consequences of relative deprivation in a non-judgmental or active manner (87). Migrant children with high belief in a just world have comprehended relative deprivation in some positive way that enabled them to accept the gap with urban peers and cope more effectively with their negative cognition and emotion (88), and thus to be less likely to experience low self-esteem. Additionally, migrant children believe that the societal issue of migration could be improved by themselves when the belief in a just world is high. Third, belief in a just world buffers against the negative effect of relative deprivation (25). Individuals with low belief in a just world are motivated to perceive relative deprivation compared with those with a high belief in a just world. In our study, relative deprivation and belief in a just world interact and mutually affect self-esteem. Thus, migrant children with high belief in a just word might avoid negative effects if they have low relative deprivation, and migrant children with high relative deprivation exacerbate unfavorable effects (e.g., low self-esteem) if they have low belief in a just world. In accordance with the model of risk and protective factors (59), belief in a just world is a protective factor and ameliorates the impact of relative deprivation. In brief, migrant children who have high belief in a just world may better handle relative deprivation and the consequent adverse consequences.

### Limitations and implications

Although several important insights were gained from our study, several limitations must also be considered. First, causal inferences among the variable associations could not be made owing to the cross-sectional survey design. Thus, future studies should conduct longitudinal or experimental research to confirm causal associations in the theoretical model. Second, the self-report method may have introduced social desirability bias and common method bias. Future research should thus collect data using multiple methods and from multiple informants. Third, some significant demographic information was neglected when we collected questionnaires, such as the ethnic conditions. Future research should take various information of migrant children into full consideration to further validate the findings of this study. Fourth, since this study was conducted on Chinese rural-to-urban migrant children, the generalization of our findings to other groups or populations from different countries may not be possible. Future studies should consider different types of participants (e.g., non-migrant children and foreign migrant children).

Despite these limitations, this study has important implications. First, this study's findings have profound theoretical implications. Based on relevant research and theories, the psychological mechanisms underlying relative deprivation and depressive symptoms were revealed. Specifically, this study explained how and when relative deprivation influenced migrant children's depressive symptoms. The present model was in line with the just-world theory (56) and risk and protective factor models (59). Furthermore, the large sample utilized in this study provides sufficient evidence for the relationship between relative deprivation and depressive symptoms. Additionally, testing mediator and moderator variables in a single model provides more comprehensive information than assessing two separate models (89).

Second, the study's results have far-reaching practical implications. Specifically, this study aimed to examine the relationship between relative deprivation and depressive symptoms and extend the relationship to a new group, namely Chinese rural-to-urban migrant children. Furthermore, given the direct association between relative deprivation and depressive symptoms, the findings highlighted that parents should help migrant children learn that relative deprivation has adverse impacts on depressive symptoms and assist them in exerting a positive role of relative deprivation (90). Meanwhile, the signs and undesirable outcomes of depressive symptoms were highlighted, which can enable educators to recognize them. Considering that self-esteem was a significant mechanism linking relative deprivation to depressive symptoms, migrant children should learn some self-reference tasks (91) and compassionate actions (92) to enhance self-esteem. Moreover, the moderating effect of belief in a just world suggests that improving migrant children's belief in a just world is an effective complementary intervention strategy to relieve relative deprivation and depressive symptoms. Research has demonstrated that belief in a just world can be cultivated and developed through intervention training and daily practice, such as psychological intervention programs (54).

## Conclusion

Although further replication and extension are needed, this study expanded the understanding of the potential mechanisms of relative deprivation on depressive symptoms in Chinese rural-to-urban migrant children by supporting the mediating role of self-esteem and the moderating role of belief in a just world. This moderated mediation model is significant because it moves beyond simple mediation and/or moderation and emphasizes how and when relative deprivation affects depressive symptoms. Moreover, these results provide a vast and compelling body of evidence that can be utilized in clinical practice. These findings can guide parents, educators, and society to take measures to improve migrant children's self-esteem and belief in a just world, thus protecting them from the detrimental effects of high level of relative deprivation and decreasing the risk of depressive symptoms.

## Data availability statement

The raw data supporting the conclusions of this article will be made available by the authors, without undue reservation.

## Ethics statement

The studies involving human participants were reviewed and approved by Ethics Committee of the Academic Research at Yangtze University. Written informed consent to participate in this study was provided by the participants' legal guardian/next of kin.

## Author contributions

MX conceived and designed the study, performed the survey, and authored and reviewed drafts of the paper. ZH analyzed the data, prepared figures and tables, and wrote it into the article. Both authors contributed to the article and approved the submitted version.

## Funding

This research was supported by grants from the Major Project for Philosophy and Social Science Research of Hubei Province (No. 19ZD020) and from the Key Project of Educational Science Planning of Hubei Province (No. 2022GA028) in China.

## Conflict of interest

The authors declare that the research was conducted in the absence of any commercial or financial relationships that could be construed as a potential conflict of interest.

## Publisher's note

All claims expressed in this article are solely those of the authors and do not necessarily represent those of their affiliated organizations, or those of the publisher, the editors and the reviewers. Any product that may be evaluated in this article, or claim that may be made by its manufacturer, is not guaranteed or endorsed by the publisher.
